# From text to video: what will OpenAI’s Sora bring to the oncologic field?

**DOI:** 10.1097/JS9.0000000000001988

**Published:** 2024-08-02

**Authors:** Wanqing Li, Jia Yang, Qipeng Shao, Haiyang Wu, Cheng Li

**Affiliations:** a Department of Operating Room, Xiangyang Central Hospital, Affiliated Hospital of Hubei University of Arts and Science, Xiangyang; b Department of Orthopaedics, Jincheng General Hospital, Jincheng, Shanxi Province; c Department of Orthopaedics, Ganzhou People’s Hospital, Ganzhou; d Department of Orthopaedics, The First Affiliated Hospital of Zhengzhou University, Zhengzhou; e Department of Spine Surgery, Wangjing Hospital, China Academy of Chinese Medical Sciences, Beijing, China; f Center for Musculoskeletal Surgery (CMSC), Charité-Universitätsmedizin Berlin, Corporate Member of Freie Universität Berlin, Humboldt University of Berlin, Berlin Institute of Health, Berlin, Germany

*Dear Editor,*

Recently, *International Journal of Surgery* has published an article about Sora^1^. Released in February 2024, Sora is OpenAI’s newest text-to-video generation model. This model is capable of producing high-fidelity video clips up to 60 s in length according to textual descriptions^2^. Sora not only surpasses existing products on the market in terms of video quality and consistency but also garners significant attention in specialized fields such as medicine^2,3^.

## Technological advantages of Sora

The advancements in text-to-image and video technologies over the past few years have paved the way for Sora’s development. Starting with the release of ChatGPT in 2022, followed by the emergence of text-to-static image technologies like Midjourney, Stable Diffusion, and DALL-E, these breakthroughs have accelerated the evolution of AI-generated videos. Although companies like Meta, Google, Runway, and Nvidia have previously explored this domain with impressive products such as Make-A-Video, Imagen, and Gen-2, Sora represents a new pinnacle in AI video generation technology^4–6^. Leveraging the combined capabilities of GPT-4 and diffusion transformer architectures, Sora exhibits remarkable prowess in natural language understanding, which enables it to interpret and render complex physical scenes with precision^7,8^. In contrast to earlier models that could generate videos up to 18 seconds in length, Sora extends this capability to produce longer, more coherent videos, reaching up to 60 s in duration. It also significantly enhances video detail and resolution, and achieve a high definition of 1920×1080 pixels, which could effectively mitigate common pixelation issues in generated videos. In addition, Sora’s strengths lie not only in its technical breakthroughs but also in its ability to accurately depict scenes and actions. It could generate videos featuring multiple characters, intricate actions, and rich background details with high temporal consistency, resulting in a visually smooth and natural video experience. These characteristics endow Sora with immense potential in generating high-precision and consistent medical videos. Here, we will for the first time explore Sora’s potential impact on the field of oncology, including applications in health education, patient-doctor communication, perioperative management, and scholarly communication.

## Health education

Health education is crucial for improving health literacy and outcomes among cancer patients. However, research indicates that there is a significant gap in health information seeking and engagement in care among these patients. Currently, less than 5% of cancer patients actively participate in their care, and between 39.7 and 54.0% exhibit low levels of proactive involvement^9^. Health literacy is fundamental in shaping patients’ health behaviors and is a critical factor in their medical decision-making processes. Therefore, enhancing the health literacy of cancer patients is of paramount importance. Several previous studies have found that short video platforms such as TikTok are effective methods for health education^10,11^. In terms of the health education of cancer patients, healthcare providers and educational teams could leverage advanced technologies such as the text-to-video model Sora. This tool could generate and disseminate specific knowledge about oncology and preventive measures. By utilizing engaging video formats, health education is able to effectively convey information about various types of tumors, their symptoms, early screening, and prevention strategies to the public. Moreover, Sora offers significant advantages in health education. It could produce culturally and linguistically tailored video content, thereby broadening the reach of educational efforts. Additionally, it translates complex medical information into more comprehensible and engaging formats, and also enhance public acceptance and understanding.

## Patient-doctor communication

In contemporary medical settings, the quality of patient-doctor communication profoundly impacts patient outcomes and satisfaction. The text-to-video model Sora facilitates this communication by generating videos that elucidate complex medical terms and diagnostic results, thereby enhancing patients’ comprehension of medical information. This approach significantly bolsters patients’ trust in the medical process. In addition, the short video format employed by Sora enables swift and effective information transfer. Oncologists could use these videos to directly explain intricate medical details, which allows patients to gain a clearer and more intuitive understanding of their conditions and treatment options. This enhanced comprehension fosters greater trust in the proposed treatment plans.

## Perioperative management

In oncological surgery, effective perioperative management is crucial for both surgical success and postoperative recovery. Sora could enhance this process by generating comprehensive preoperative preparation videos. These videos provide patients with a clear understanding of the surgical process, important considerations, and necessary preoperative preparations, which could significantly reduce pre-surgical anxiety and apprehension. For instance, one previous study investigated whether showing information videos in patients’ first languages, explaining spinal anesthesia for cesarean delivery, could alleviate anxiety, improve satisfaction, and enhance doctor-patient communication in the presence of language barriers^12^. The results demonstrated that patients who watched the information video had significantly lower Numerical Visual Analog Anxiety Scale (NVAAS) scores compared to those receiving standard care. Moreover, Sora allows oncologists to create personalized postoperative care videos covering dietary recommendations, rehabilitation exercises, and medication instructions. These tailored videos offer specific guidance, reminding patients of each postoperative step and consideration, thereby facilitating better postoperative recovery and accelerating the rehabilitation process.

## Scholarly communication

Traditionally, academic findings have predominantly been disseminated through text-based publications in specialized journals. While this conventional method excels in rigor and authority, it suffers from slow dissemination speeds and a limited audience reach. In recent years, the advent of various short video platforms has prompted numerous journal publishers to encourage authors to convert their textual content into video format. This shift not only diversifies the modalities of academic communication but also broadens the potential audience. Text-to-video generation models have introduced a novel tool for the medical community. The application of Sora technology enables researchers to visualize their written descriptions of research findings, experimental procedures, and data, transforming them into concise and comprehensible video presentations. Additionally, leveraging text-to-video tools to swiftly create academic reports and explanatory videos could make academic conferences more dynamic and accessible. Sora could convert intricate academic content into engaging videos, utilizing charts, animations, and other visual aids to clearly present research data and conclusions, significantly improving the efficiency and effectiveness of scholarly communication.

## Future challenges

Despite the impressive performance of Sora, it still faces several challenges. For instance, the model needs further improvement in simulating the physical principles of complex scenarios, understanding causal relationships, and capturing spatial details. While these issues may be mitigated in the short term through enhanced computational power and model refinement, other challenges associated with AI-generated video technology, such as copyright ownership, data security, and patient privacy, require more comprehensive guidelines and even legal regulations. In the field of oncology, although these text-to-video tools hold great promise, AI-generated videos must be based on accurate data and models to prevent misdiagnoses or incorrect treatment plans, which could have severe consequences for patients. Furthermore, the issue of copyright ownership of AI-generated videos remains complex. When an AI-generated video is used for medical research or publication, defining intellectual property rights becomes a challenging task yet to be resolved. Finally, the application of AI-generated video technology in the medical field also raises concerns about patient privacy and data security. If these videos contain sensitive patient information, it is crucial to ensure data protection and anonymization to prevent privacy breaches. Additionally, the use and sharing of medical data must strictly comply with relevant laws and regulations to avoid any form of misuse or infringement.

In summary, Sora’s innovation and breakthrough in the field of text-to-video generation are undoubtedly remarkable, and its significant improvement in quality and consistency points the way for the development of future AI video generation technology. Through this study, we hope to introduce Sora’s technical advantages and development prospects to readers, while also exploring its application potential and risks in the field of oncology.

## Source of funding

This study is supported by China Postdoctoral Science Foundation (2022M720385) and Beijing JST Research Funding (YGQ-202313).

## Author contribution

W.L., J.Y., and Q.S.: conceptualization, methodology, data curation, formal analysis, resources, investigation, writing—original draft; H.W. and C.L.: conceptualization, methodology, formal analysis, resources, investigation, writing—review and editing.

## Conflicts of interest disclosure

The authors declare no conflict of interest.

## Research registration unique identifying number (UIN)

1. Name of the registry:
2. Unique Identifying number or registration ID:
3. Hyperlink to your specific registration (must be publicly accessible and will be checked):

## Guarantor

Haiyang Wu, Cheng Li.

## Data availability statement

The data underlying this article will be shared by the corresponding author on reasonable request.
